# What do Cochrane systematic reviews say about non-pharmacological interventions for treating cognitive decline and dementia? O que as revisões sistemáticas Cochrane falam sobre intervenções não farmacológicas para o tratamento de declínio cognitivo e demência?

**DOI:** 10.1590/1516-3180.2017.0092060617

**Published:** 2017-04-03

**Authors:** Vitória Carvalho Vilela, Rafael Leite Pacheco, Carolina Oliveira Cruz Latorraca, Daniela Vianna Pachito, Rachel Riera

**Affiliations:** I Undergraduate Medical Student, Escola Paulista de Medicina (EPM), Universidade Federal de São Paulo (UNIFESP), São Paulo (SP), Brazil.; II Master and Psychologist, Evidence-Based Health Program, Universidade Federal de São Paulo (UNIFESP), São Paulo (SP), Brazil; and Assistant Researcher, Cochrane Brazil, São Paulo (SP), Brazil.; III Master of Science, Neurologist and Doctoral Student, Evidence-Based Health Program, Universidade Federal de São Paulo (UNIFESP), São Paulo (SP), Brazil; and Assistant Researcher, Cochrane Brazil, São Paulo (SP), Brazil.; IV Doctor and Master of Science, Rheumatologist and Adjunct Professor, Discipline of Evidence-Based Medicine, Escola Paulista de Medicina (EPM), Universidade Federal de São Paulo (UNIFESP); and Assistant Coordinator, Cochrane Brazil, São Paulo (SP), Brazil.

**Keywords:** Dementia, Alzheimer disease, Review, Evidence-based practice, Evidence-based medicine

## Abstract

**BACKGROUND:**

Dementia is a highly prevalent condition worldwide. Its chronic and progressive presentation has an impact on physical and psychosocial characteristics and on public healthcare. Our aim was to summarize evidence from Cochrane reviews on non-pharmacological treatments for cognitive disorders and dementia.

**DESIGN AND SETTING:**

Review of systematic reviews, conducted in the Discipline of Evidence-Based Medicine, Escola Paulista de Medicina, Universidade Federal de São Paulo.

**METHODS:**

Cochrane reviews on non-pharmacological interventions for cognitive dysfunctions and/or type of dementia were included. For this, independent assessments were made by two authors.

**RESULTS:**

Twenty-four reviews were included. These showed that carbohydrate intake and validation therapy may be beneficial for cognitive disorders. For dementia, there is a potential benefit from physical activity programs, cognitive training, psychological treatments, aromatherapy, light therapy, cognitive rehabilitation, cognitive stimulation, hyperbaric oxygen therapy in association with donepezil, functional analysis, reminiscence therapy, transcutaneous electrical stimulation, structured decision-making on feeding options, case management approaches, interventions by non-specialist healthcare workers and specialized care units. No benefits were found in relation to enteral tube feeding, acupuncture, Snoezelen stimulation, respite care, palliative care team and interventions to prevent wandering behavior.

**CONCLUSION:**

Many non-pharmacological interventions for patients with cognitive impairment and dementia have been studied and potential benefits have been shown. However, the strength of evidence derived from these studies was considered low overall, due to the methodological limitations of the primary studies.

## RESUMO

**INTRODUÇÃO:**

Demência é uma condição com alta prevalência e incidência global. Sua característica crônica e progressiva tem impacto em aspectos físicos, psicossociais e na saúde pública. Nosso objetivo foi resumir evidências de revisões Cochrane sobre intervenções não farmacológicas para distúrbios cognitivos e demências.

**TIPO DE ESTUDO E LOCAL:**

Revisão de revisões sistemáticas conduzida na Disciplina de Medicina Baseada em Evidências da Escola Paulista de Medicina, Universidade Federal de São Paulo.

**MÉTODOS:**

Foram incluídas revisões Cochrane sobre intervenções não farmacológicas para disfunções cognitivas e/ou qualquer tipo de demência, após a avaliação realizada de forma independente por dois autores.

**RESULTADOS:**

Vinte e quatro revisões foram incluídas. As revisões mostraram que ingestão de carboidratos e a terapia de validação podem ser benéficas para distúrbios cognitivos. Para demência, existe benefício potencial de programas de atividade física, treino cognitivo, tratamentos psicológicos, aromaterapia, terapia com luz, reabilitação cognitiva, estimulação cognitiva, oxigenoterapia hiperbárica associada a donepezila, análise funcional, terapia de reminiscência, estimulação elétrica transcutânea, decisão estruturada em opções de alimentação, abordagem de gestão de casos e intervenções aplicadas por trabalhadores na área de saúde não especialistas e por unidades de cuidado especializado. Não foram encontrados benefícios para alimentação por sonda entérica, acupuntura, estimulação de Snoezelen, cuidados de repouso, equipe de cuidados paliativos e intervenções para prevenir comportamento de perambulação.

**CONCLUSÃO:**

Várias intervenções não farmacológicas para pacientes com comprometimento cognitivo e demência têm sido estudadas, mostrando benefícios potenciais. Entretanto, a força de evidência derivada desses estudos é em geral considerada baixa, devido às limitações metodológicas dos estudos primários.

## INTRODUCTION

Dementia has been considered by the World Health Organization to be a public health priority since 2012,^1^ because of its high estimated prevalence and incidence. A report published in 2016 estimated that 47.5 million people have dementia worldwide and this number is expected to almost triple by 2050, to reach 135.5 million.^2^ Because of the chronic and progressive nature of this condition, the socioeconomic impact of dementia is extremely important. As a consequence of the estimated increase in prevalence, increases in the familial and societal burden and an even more significant impact on healthcare costs can be expected.^3^^,^^4^

Treatment and management of dementia are challenging because of the patients’ diminished ability to adhere to therapeutics and to report adverse effects.^5^ Also, dementia is not a ­single condition: rather, it comprises distinct diseases with ­different pathophysiological mechanisms. The therapeutic and preventive strategies depend particularly on understanding ­the ­etiology and other factors such as ­clinical features, stage of dementia and family support. Some of the most common pharmacological agents are cholinesterase inhibitors,^6^ ­memantine,^7^ memantine combined with ­cholinesterase ­inhibitors^8^^,^^9^ and antioxidants.^10^^,^^11^ There are many non-pharmacological ­therapies and some of them show little, if any, evidence of benefit regarding dementia.

A quick search on MEDLINE (via PubMed), using the MeSH term “dementia” and applying a filter to identify clinical trials*,* retrieved an average of 268 (153-334) published papers per year over the last 10 years. The high number of studies published over recent years and the clinical importance of this issue have provided the impulse for comprehensive research syntheses such as this review of reviews.

## OBJECTIVES

To identify and summarize Cochrane systematic reviews ­focusing on non-pharmacological interventions to treat ­cognitive impairment and dementia, regardless of etiology, and to present their findings in accordance with the quality of the evidence.

## METHODS

### Design

Review of Cochrane systematic reviews on interventions to treat cognitive impairment and dementia. 

### Setting

Discipline of Evidence-Based Medicine of Escola Paulista de Medicina, Universidade Federal de São Paulo (UNIFESP).

### Criteria for including reviews

• Types of studies 

We included the latest version of completed Cochrane systematic reviews, without imposing any restriction on publication date. Protocols relating to systematic reviews and reviews that were coded as “withdrawn” in the Cochrane Database of Systematic Reviews (CDSR) were not included. 

• Types of participants

Patients diagnosed with cognitive impairment or dementia, regardless of etiology, including (but not limited to) mild cognitive impairment, vascular dementia, Alzheimer, mixed dementia and dementia secondary to other neurodegenerative diseases.

• Types of intervention

Non-pharmacological interventions including (but not limited to) psychological, social and educational interventions, acupuncture, physical exercise and physical therapy. 

• Types of outcomes 

Clinical, social and laboratory outcomes, as reported in the systematic reviews.

### Search for reviews 

We conducted a systematic search in the Cochrane Database of Systematic Reviews (CDSR) (via Wiley) on December 19, 2016, using a sensitive search strategy (**Table 1**).

**Table 1:**
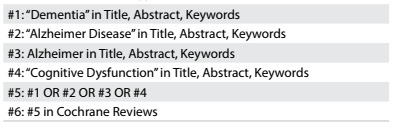
Search strategy (December 19, 2016)

### Selection of systematic reviews 

Two reviewers independently evaluated titles and abstracts of records initially retrieved on the basis of the inclusion criteria. Disagreements were solved by reaching a consensus.

### Presentation of the results

We presented the results from the systematic reviews included through a narrative structure (qualitative synthesis).

## RESULTS

### Search results

The initial search retrieved 183 reviews. However, only 24 fulfilled our inclusion criteria.^12^^,^^13^^,^^14^^,^^15^^,^^16^^,^^17^^,^^18^^,^^19^^,^^20^^,^^21^^,^^22^^,^^23^^,^^24^^,^^25^^,^^26^^,^^27^^,^^28^^,^^29^^,^^30^^,^^31^^,^^32^^,^^33^^,^^34^^,^^35^

### Results from systematic reviews

Among the 24 systematic reviews included, two (~8%) focused on vascular dementia, two (~8%) focused on dementia secondary to other diseases and 16 (~66.6%) focused on all types of dementia. Additionally, one study (~4%) focused on cognition as a broader topic, two (~8%) focused on both dementia and mild cognitive impairment and one (~4%) on mental disorders as a broader topic. Three reviews (12.5%) assessed caregivers’ outcomes, and six (25%) focused on interventions relating to healthcare systems.

A brief summary of the systematic reviews included is presented below. The issues addressed, the main findings from each intervention and the quality of the evidence (based on the GRADE approach) are presented in **Tables 2**, 3 and 4. ^12^^,^^13^^,^^14^^,^^15^^,^^16^^,^^17^^,^^18^^,^^19^^,^^20^^,^^21^^,^^22^^,^^23^^,^^24^^,^^25^^,^^26^^,^^27^^,^^28^^,^^29^^,^^30^^,^^31^^,^^32^^,^^33^^,^^34^^,^^35^^,^^36^

**Table 2:**
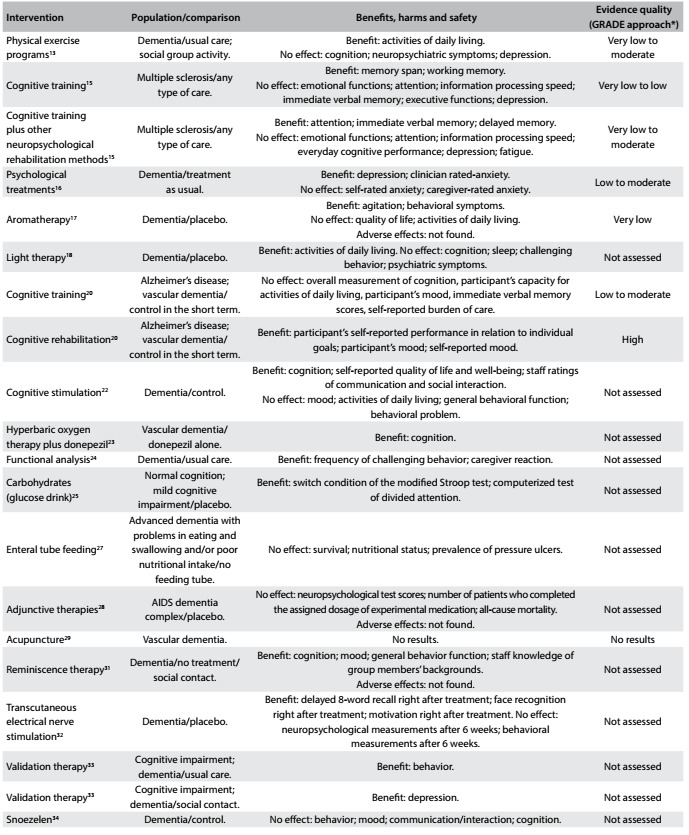
Characteristics, main findings and quality of evidence from systematic reviews focusing on patient-directed interventions

*GRADE = Grading of Recommendations Assessment, Development and Evaluation. Tool used for assessing the quality of the body of the current evidence. High quality: low probability that further studies might change the confidence regarding the existing evidence. Moderate quality: probability that further studies will change the confidence regarding the existing evidence. Low quality: high probability that further studies will change the confidence regarding the existing evidence. Very low quality: there is much uncertainty about the information, precluding any valid interpretation.^36^

**Table 3:**
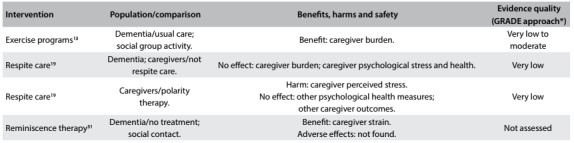
Characteristics, main findings and quality of evidence from systematic reviews focusing on caregiver-directed interventions

*GRADE = Grading of Recommendations Assessment, Development and Evaluation. Tool used for assessing the quality of the body of the current evidence. High quality: low probability that further studies might change the confidence regarding the existing evidence. Moderate quality: probability that further studies will change the confidence regarding the existing evidence. Low quality: high probability that further studies will change the confidence regarding the existing evidence. Very low quality: there is much uncertainty about the information, precluding any valid interpretation.^36^

**Table 4:**
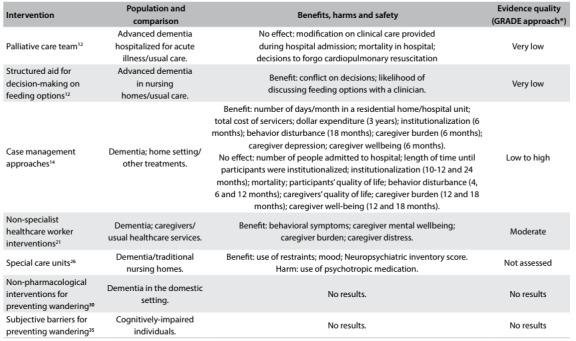
Characteristics, main findings and quality of evidence from systematic reviews focusing on healthcare system interventions

*GRADE = Grading of Recommendations Assessment, Development and Evaluation. Tool used for assessing the quality of the body of the current evidence. High quality: low probability that further studies might change the confidence regarding the existing evidence. Moderate quality: probability that further studies will change the confidence regarding the existing evidence. Low quality: high probability that further studies will change the confidence regarding the existing evidence. Very low quality: there is much uncertainty about the information, precluding any valid interpretation.^36^

#### 1. Palliative care interventions 

The review^12^ (2016) had the purpose of assessing the effectiveness of palliative care interventions in cases of advanced dementia, with inclusion of two randomized controlled trials (RCTs). Because of the heterogeneity of the data, no meta-analysis could be done. The results listed below were found:

- Palliative care team for people with advanced dementia hospitalized for an acute illness (99 participants): no evidence of in-hospital mortality [risk ratio (RR) 1.06, 95% confidence interval (CI) 0.53 to 2.13], cardiopulmonary resuscitation, or clinical care provided during hospital admission;
- Structured decision-making aid for feeding options among surrogate decision-makers of nursing-home residents with advanced dementia (total of 90 participants included): lower scores for decisional conflict [mean difference (MD) -0.30, 95% CI -0.61 to 0.01] in the group of intervention surrogates and more likelihood of discuss feeding options with a clinician, in comparison with the control group (RR 1.57, 95% CI 0.93 to 2.64).

There was insufficient evidence to assess the effect of palliative care interventions on advanced dementia. For further details, refer to the original abstract, available at: http://onlinelibrary.wiley.com/doi/10.1002/14651858.CD011513.pub2/abstract.

### 2. Physical exercise programs 

The objective of the review^13^ (2015) was to analyze physical exercise programs for dementia on different outcomes, such as cognition, activities of daily living, neuropsychiatric symptoms, depression and mortality, and the secondary outcomes from the intervention (on family caregivers and on use of healthcare services). A meta-analysis on 17 trials (1067 participants) showed the following results (exercise versus usual care/social group activity):

- Activities of daily living: benefit from exercise programs [six ­trials, 289 participants; standardized mean difference (SMD) 0.68; 95% CI 0.08 to 1.27, P = 0.02);
- Cognitive functioning: no clear evidence (nine studies, 409 participants; SMD 0.43; 95% CI -0.05 to 0.92, P = 0.08);
- Neuropsychiatric symptoms and depression: no clear evidence. For neuropsychiatric symptoms: one trial, 110 participants; MD -0.60; 95% CI -4.22 to 3.02; P = 0.75; for depression: five trials, 341 participants; SMD 0.14; 95% CI -0.07 to 0.36; P = 0.16;
- Caregiver burden: may be reduced when the caregiver supervises the patient in an exercise program (one trial, 40 participants; MD -15.30; 95% CI -24.73 to -5.87; P = 0.001);
- Other secondary outcomes could not be assessed.

There was promising evidence that exercise programs might improve the ability of people with dementia to perform activities of daily living, but no evidence of benefit regarding cognition, neuropsychiatric symptoms, or depression. For further details, refer to the original abstract, available at: http://onlinelibrary.wiley.com/doi/10.1002/14651858.CD006489.pub4/abstract.

#### 3. Case management approaches to home support 

Case management is an intervention for organizing and coordinating care at the level of the individual, providing long-term care for people with dementia in the community. The aim of the review^14^ (2015) was to evaluate the effect of case management approaches to home support for dementia, from the perspectives of the different parties involved (patients, caregivers and staff). It included 13 RCTs (9615 participants). The following results were found (comparison of case management versus other treatments):

- Total cost of services: reduction in the case management group at 12 months (two RCTs, n = 5,276; SMD -0.07, 95% CI -0.12 to -0.02, P = 0.01);
- Dollar expenditure: reduction for the total of three years (one RCT, n = 5170; MD -705.00, 95% CI -1170.31 to -239.69, P = 0.003);
- Number of days per month in a residential home or hospital unit: reduction in the case management group at six months (one RCT, n = 88; MD -5.80, 95% CI -7.93 to -3.67, P < 0.0001) and at 12 months (one RCT, n = 88; MD -7.70, 95% CI -9.38 to -6.02, P < 0.0001);
- Number of people admitted to hospital: no differences at six months (four RCTs, 439 participants), 12 months (five RCTs, 585 participants) and 18 months (five RCTs, 613 participants);
- Length of time until participants were institutionalized: uncertain effects at 12 months (one trial; hazard ratio (HR): 0.66, 95% CI 0.38 to 1.14, P = 0.14);
- Institutionalization (admission to residential or nursing homes): significantly less likely for case management group at six months (six RCTs, n = 5741; OR 0.82, 95% CI 0.69 to 0.98, I² = 0%, P = 0.02) and at 18 months (four RCTs, n = 363; OR 0.25, 95% CI 0.10 to 0.61, I² = 0%, P = 0.003). The effects were uncertain at 10 to 12 months (nine RCTs, n = 5990; OR 0.95, 95% CI 0.83 to 1.08, I² = 48%, P = 0.39) and at 24 months (two RCTs, n = 201; OR 1.03, 95% CI 0.52 to 2.03, I² = 0%, P = 0.94);
- Mortality and participants’ or caregivers’ quality of life: no significant events (mortality: at four, six, 12, 18, 24 and 36 months; quality of life: at four, six, 12 and 18 months);
- Behavioral disorder: reduction in case management group at 18 months (2 RCTs, n = 206; SMD -0.35, 95% CI -0.63 to -0.07, I² = 0%, P = 0.01) but uncertain effects at four months (two RCTs), six months (four RCTs) and 12 months (five RCTs);
- Caregiver burden: benefits at six months (four RCTs, n = 4601; SMD -0.07, 95% CI -0.12 to -0.01, I² = 26%, P = 0.03) but uncertain effects at 12 or 18 months;
- Caregiver depression: small significant improvement in case management group at 18 months (three RCTs, n = 2,888; SMD -0.08, 95% CI -0.16 to -0.01, I² = 0%, P = 0.03);
- Caregiver wellbeing: greater improvement in the case management group at six months (one RCT, n = 65; MD -2.20 CI -4.14 to -0.26, P = 0.03) but uncertain effects at 12 or 18 months.

There was evidence that case management was beneficial for improving some outcomes relating to patients and caregivers and for lowering admissions to care homes and overall healthcare costs. There was not enough evidence regarding whether case management might delay institutionalization in care homes, and there were uncertain results regarding patient depression, functional abilities and cognition. For further details, refer to the original abstract, available at: http://onlinelibrary.wiley.com/doi/10.1002/14651858.CD008345.pub2/abstract.

#### 4. Neuropsychological rehabilitation on multiple sclerosis

Cognitive deficits are a common manifestation of multiple sclerosis (MS). The review^15^ (2014) aimed to assess the effects of neuropsychological/cognitive rehabilitation on health-related factors (cognitive performance and emotional well-being) among patients with MS, and included 20 studies (986 participants, mean age of 44.6 years and 70% women). The results are listed below (comparison: intervention versus control):

- Cognitive training: improvement of memory span (SMD 0.54, 95% CI 0.20 to 0.88, P = 0.002) and of working memory (SMD 0.33, 95% CI 0.09 to 0.57, P = 0.006). No evidence of effect on emotional functions, attention, information processing speed, immediate verbal memory, executive functions or depression;
- Cognitive training combined with other neuropsychological rehabilitation methods: improvement of attention (SMD 0.15, 95% CI 0.01 to 0.28, P = 0.03), of immediate verbal memory (SMD 0.31, 95% CI 0.08 to 0.54, P = 0.008) and of delayed memory (SMD 0.22, 95% CI 0.02 to 0.42, P = 0.03). No evidence of effect on emotional function, attention, information processing speed, everyday cognitive performance, depression or fatigue.

The review found low-level evidence of positive effects from neuropsychological rehabilitation in relation to MS. For further details, refer to the original abstract, available at: http://onlinelibrary.wiley.com/doi/10.1002/14651858.CD009131.pub3/abstract.

#### 5. Psychological treatments 

Anxiety and depressive symptoms are very common in cases of dementia and mild cognitive impairment. The purpose of the review^16^ (2014) was to assess the effectiveness of psychological interventions (cognitive behavioral therapy, interpersonal therapy, counselling and others) on anxiety and depression in cases of dementia and mild cognitive impairment. It included six RCTs on dementia (439 participants, six to 12 months of intervention). No studies focusing on mild cognitive impairment were included. The results listed below were found (comparison: psychological treatment versus treatment as usual):

- Depression: positive effect from psychological treatments (six trials, 439 participants; SMD -0.22, 95% CI -0.41 to -0.03);
- Clinician-rated anxiety: positive effect from ­psychological treatments (two trials, 65 participants; MD -4.57, 95% CI -7.81 to -1.32);
- Self-rated and caregiver-rated anxiety: no difference (for self-rated: two trials, SMD 0.05, 95% CI -0.44 to 0.54; for caregiver-rated: one trial, MD -2.40, 95% CI -4.96 to 0.16).

There was evidence that psychological interventions combined with usual care could reduce the symptoms of depression and clinician-rated anxiety among people with dementia. For further details, refer to the original abstract, available at: http://onlinelibrary.wiley.com/doi/10.1002/14651858.CD009125.pub2/abstract.

#### 6. Aromatherapy 

The objective of the review^17^ (2014) was to assess the effectiveness of aromatherapy for treating dementia. Seven RCTs (428 participants, three to 12 weeks of intervention) were included in this review, but only two were combined in a meta-analysis. The results listed below were found (comparison: aromatherapy versus placebo):

- Agitation and behavioral symptoms: significant treatment effect from aromatherapy (one study; for agitation: n = 71, MD -11.1, 95% CI -19.9 to -2.2; for behavioral symptoms: n = 71, MD -15.8, 95% CI -24.4 to -7.2) versus no difference (one study; for agitation: n = 63, MD 0.00, 95% CI -1.36 to 1.36; for behavioral symptoms: n = 63, MD 2.80, 95% CI -5.84 to 11.44);
- Quality of life and activities of daily living: no difference in the comparison (one study; for quality of life: n = 63, MD 19.00, 95% CI -23.12 to 61.12; for activities of daily living: n = 63, MD -0.50, 95% CI -1.79 to 0.79);
- Adverse effects: no difference (two studies, n = 124; RR 0.97, 95% CI 0.15 to 6.46).

The benefits of aromatherapy for people with dementia were equivocal according to this review. For further details, refer to the original abstract, available at: http://onlinelibrary.wiley.com/doi/10.1002/14651858.CD003150.pub2/abstract.

#### 7. Light therapy

Stimulation of suprachiasmatic nuclei using light might have the potential to reverse circadian disturbances in cases of dementia. The review^18^ (2014) examined the effect of light therapy on cognition, activities of daily living, sleep, challenging behavior and psychiatric symptoms associated with dementia, and included 11 trials (499 participants), among which only 8 could be combined in a meta-analysis. The following results were found (­comparison: light therapy versus placebo):

- Activities of daily living: reduction in the development of limitations (one study);
- Cognitive function, sleep, challenging behavior or psychiatric symptoms associated with dementia: no effect.

There was insufficient evidence to justify the use of bright-light therapy in cases of dementia. For further details, refer to the original abstract, available at: http://onlinelibrary.wiley.com/doi/10.1002/14651858.CD003946.pub4/abstract.

#### 8. Respite care 

Respite care is any intervention designed to give rest or relief to caregivers. The review^19^ (2014) aimed to assess the effect of respite care on dementia patients and their caregivers, particularly regarding institutionalization rates. Four trials (753 participants) were included, but no meta-analysis could be done. The following results were found:

- Respite care versus no respite care: no significant effects on caregiver variables (burden and psychological stress and health);
- Respite care versus polarity therapy: significant effect found in favor of polarity therapy for caregiver-perceived stress (n = 38, MD 5.80, 95% CI 1.43 to 10.17), but not for other psychological health measures and other caregiver outcomes;
- Outcomes for people with dementia: not reported in the studies.

The current evidence did not demonstrate any benefits or adverse effects from the use of respite care, for people with dementia or their caregivers. For further details, refer to the original abstract, available at: http://onlinelibrary.wiley.com/doi/10.1002/14651858.CD004396.pub3/abstract.

#### 9. Cognitive training and cognitive rehabilitation 

Cognitive training and cognitive rehabilitation are interventions for improving memory and other aspects of cognitive functioning. The review^20^ (2013) aimed to evaluate the effectiveness of these two types of interventions for Alzheimer’s disease or vascular dementia, including 11 trials (383 participants receiving interventions from four to 24 weeks) on cognitive training and one on rehabilitation. The following results were found (comparison: intervention versus control over the short term):

- Cognitive training: no effect on any outcomes (overall measurement of cognition, participant’s capacity for activities of daily living, participant’s mood, immediate verbal memory scores and self-reported burden of care);
- Cognitive rehabilitation: no meta-analysis could be conducted. However, promising results were found for other outcomes (participant’s self-reported performance in relation to individual goals, participant’s mood and self-reported mood).

This review did not provide evidence to confirm that cognitive training is effective. Although the results regarding cognitive rehabilitation were positive, they were considered preliminary in nature. For further details, refer to the original abstract, available at: http://onlinelibrary.wiley.com/doi/10.1002/14651858.CD003260.pub2/abstract.

#### 10. Interventions to care for mental disorders, conducted by non-specialist healthcare workers 

A significant number of people suffering from mental, neurological and substance-use disorders do not receive adequate healthcare. Use of non-specialist healthcare workers and other professionals involved in healthcare is a key strategy for closing the treatment gap. The objective of the review^21^ (2013) was to assess the effectiveness of non-specialist healthcare workers and other professionals involved in healthcare in delivering interventions relating to mental, neurological and substance-use disorders within primary and community healthcare, in low and middle-income countries. Thirty-eight studies were included, among which 22 involved use of lay healthcare workers. For the purposes of our study, results from populations other than dementia patients are not shown (comparison: non-specialist healthcare workers versus usual healthcare services):

- Behavioral symptoms of dementia: improvement for the intervention group (severity of behavioral symptoms: SMD -0.26, 95% CI -0.60 to 0.08);
- Mental wellbeing, burden and distress of caregivers of people with dementia: improvement for the intervention group (caregiver burden: SMD -0.50, 95% CI -0.84 to -0.15).

Use of non-specialist healthcare workers and teachers provided some promising benefits in relation to improving patient and caregiver outcomes in cases of dementia. For further details, refer to the original abstract, available at: http://onlinelibrary.wiley.com/doi/10.1002/14651858.CD009149.pub2/abstract.

#### 11. Cognitive stimulation 

Cognitive stimulation (CS) includes implementation of enjoyable activities that provide general stimulation for thinking, concentration and memory. The purpose of the review^22^ (2012) was to evaluate the effects of CS on cognition in cases of dementia, and included 15 RCTs (718 patients). The following results were found (comparison: CS versus control):

- Cognitive function: benefit from CS even three months after the treatment (SMD 0.41, 95% CI 0.25 to 0.57);
- Self-reported quality of life and well-being: benefit from CS (SMD 0.38, 95% CI: 0.11, 0.65);
- Staff ratings of communication and social interaction: benefit from CS (SMD 0.44, 95% CI 0.17 to 0.71);
- Mood, activities of daily living, general behavioral function and behavioral problems: no effect.

There was consistent evidence that cognitive stimulation benefited cognition in cases of dementia. For further details, refer to the original abstract, available at: http://onlinelibrary.wiley.com/doi/10.1002/14651858.CD005562.pub2/abstract.

#### 12. Hyperbaric oxygen therapy for treating vascular dementia

Hyperbaric oxygen therapy (HBOT) has shown possible efficacy for treating vascular dementia. The aim of the review^23^ (2012) was to assess the effectiveness and safety of HBOT in treating vascular dementia, alone or as an adjuvant treatment. One study (64 patients) was included, showing the results below (comparison: HBOT plus donepezil versus donepezil alone):

- Cognitive function: benefits for the group receiving HBOT plus donepezil, which showed improvements after 12 weeks of treatment (Mini-Mental State Examination: weighted mean difference (WMD) 3.50; 95% CI 0.91 to 6.09; Hasegawa’s Dementia Rating Scale: WMD 3.10; 95% CI 1.16 to 5.04);
- Other outcomes and adverse effects: not measured in this study.

There was insufficient evidence to support HBOT as an effective treatment for patients with vascular disease. For further details, refer to the original abstract, available at: http://onlinelibrary.wiley.com/doi/10.1002/14651858.CD009425.pub2/abstract.

#### 13. Functional analysis 

Functional analysis (FA) is a promising behavioral intervention that involves exploring the meaning or purpose of an individual’s behavior. The review^24^ (2012) had the objective of assessing the effects of FA-based interventions relating to people with dementia and their caregivers. It included 18 trials, of which 14 included FA embedded in a broad multicomponent care program, which made it impossible to establish the effect of FA itself. The results showed (comparison: care program with FA versus usual care) that, for the frequency of challenging behavior and caregiver reaction, positive effects after the intervention were not assessed at the follow-up phase. The findings suggested that FA embedded in multicomponent interventions potentially had beneficial effects, but that it was precipitous to draw conclusions about its effectiveness. For further details, refer to the original abstract, available at: http://onlinelibrary.wiley.com/doi/10.1002/14651858.CD006929.pub2/abstract.

#### 14. Carbohydrates for cognition 

Carbohydrates are essential and easily accessible macronutrients that influence cognitive performance. The aim of the review^25^ (2011) was to assess the effect of carbohydrates on cognitive function in situations of normal cognition and mild cognitive impairment. One study (44 adults, aged 60 to 80 years) was included and the following results were found (comparison: glucose drink versus placebo, drunk on a single occasion):

- Switch condition of the modified Stroop test: glucose drinkers were significantly faster (F 1, 41 = 10.47; P < 0.01);
- Computerized test on divided attention: participants in the glucose group showed significantly lower dual-task cost (F 1, 38 = 8.49; P < 0.01, ² = 0.18).

There was insufficient evidence to base any recommendations regarding use of any form of carbohydrate for enhancing cognitive performance. For further details, refer to the original abstract, available at: http://onlinelibrary.wiley.com/doi/10.1002/14651858.CD007220.pub2/abstract.

#### 15. Special care units for behavioral problems 

The purpose of special care units (SCUs) is to optimize care for dementia patients, particularly those with behavioral disorders. The review^26^ (2009) aimed to evaluate the effect of SCUs on behavioral problems, mood, need for use of restraints and use of psychotropics in treating dementia. Since no RCTs met the inclusion criteria, eight non-RCTs were selected, among which only four could be combined in a meta-analysis. The following results were found (comparison: SCUs versus traditional nursing home):

- Need for use of restraints: less need for use of restraints in SCUs after six months (two studies, OR 0.46, 95% CI 0.27 to 0.80, P = 0.006) and 12 months (one study, OR 0.49, 95% CI 0.27 to 0.88, P = 0.02);
- Mood: reduction of depressive symptoms among patients at SCUs after three months (one study, WMD -6.30 (-7.88 to -4.72) Cornell points, P < 0.00001);
- Neuropsychiatric inventory score: limited improvements for patients at SCUs (one study lasting six, 12 and 18 months);
- Use of psychotropic medication: reduced use in traditional nursing home after six months (one study, WMD 0.20, CI 0.00 to 0.40, z = 1.96, P = 0.05);
- Behavioral symptoms: no studies found.

There was no strong evidence of benefit, considering the results from non-RCTs. For further details, refer to the original abstract, available at: http://onlinelibrary.wiley.com/doi/10.1002/14651858.CD006470.pub2/abstract.

#### 16. Enteral tube feeding 

Use of enteral tube feeding for patients with advanced dementia with poor nutritional intake is a frequent practice. The review^27^ (2009) aimed to evaluate enteral tube nutrition for patients with advanced dementia with eating and swallowing difficulties and/or poor nutritional intake. Seven observational controlled studies were identified. In the comparison of feeding tube versus no feeding tube, survival, nutritional status and prevalence of pressure ulcers, there was no evidence of benefit among patients receiving enteral tube feeding.

There was insufficient evidence to suggest that enteral tube feeding was beneficial among patients with advanced dementia. For further details, refer to the original abstract, available at: http://onlinelibrary.wiley.com/doi/10.1002/14651858.CD007209.pub2/abstract.

#### 17. Adjunctive therapies for treating AIDS dementia complex 

AIDS dementia complex is a complication from human immunodeficiency virus type 1. The review^28^ (2008) had the aim of determining the efficacy and safety of adjunctive therapies for treating AIDS dementia complex. Ten trials were included (711 participants). The results are shown below (comparison: 10 different treatments versus placebo):

- Neuropsychological test scores, number of patients who completed the assigned dosage of experimental medication and all-cause mortality: no significant differences between groups;
- Adverse effects: no difference between groups.

This study confirmed that there was no evidence that adjunctive therapies improved cognitive performance or quality of life among patients with AIDS dementia complex. For further details, refer to the original abstract, available at: http://onlinelibrary.wiley.com/doi/10.1002/14651858.CD006496.pub2/abstract.

#### 18. Acupuncture for treating vascular dementia 

Use of different acupuncture techniques for treating vascular dementia is an accepted practice in China. The aim of the review^29^ (2007) was to assess the effects of acupuncture therapy for treating vascular dementia. In the absence of any suitable RCTs in this field, the authors were unable to perform a meta-analysis. Therefore, the effectiveness of acupuncture for treating vascular dementia is highly uncertain. For further details, refer to the original abstract, available at: http://onlinelibrary.wiley.com/doi/10.1002/14651858.CD004987.pub2/abstract.

#### 19. Non-pharmacological interventions for preventing wandering 

Although there seems to be a consensus in the literature that, in the majority of cases, non-pharmacological approaches for preventing wandering may work as well as drug treatment and with fewer side effects, clinicians often resort to drugs as the first-line treatment for this condition. The aim of the review^30^ (2007) was to evaluate the effect and safety of non-pharmacological interventions to prevent wandering among people with dementia (domestic setting). No suitable trials on this subject were found, and therefore no results could be reported. For further details, refer to the original abstract, available at: http://onlinelibrary.wiley.com/doi/10.1002/14651858.CD005994.pub2/abstract.

#### 20. Reminiscence therapy 

Reminiscence therapy (RT) involves discussion of past activities, events and experiences, with another person or group of people and usually with the aid of representative elements, such as photos and music. The objective of the review^31^ (2005) was to assess the effects of this therapy on patients with dementia and their caregivers. It included five trials, among which only four (144 participants) presented extractable data. The results are listed below (comparison: intervention versus no treatment/social contact):

- Cognition (at follow-up), mood (at follow-up) and general behavioral function (at the end of the intervention period): statistically significant benefit;
- Caregiver strain: significant decrease for caregivers participating in groups with their relative affected by dementia;
- Staff knowledge of group members’ backgrounds: significantly improvement;
- Harmful effects: not identified.

In view of the limited number and low quality of studies, ­the ­variation in types of reminiscence work reported and the variation in results between studies, no robust conclusions could be drawn. For further details, refer to the original abstract, available at: http://onlinelibrary.wiley.com/doi/10.1002/14651858.CD001120.pub2/abstract.

#### 21. Transcutaneous electrical nerve stimulation 

Transcutaneous electrical nerve stimulation (TENS), which consists of application of electrical current through electrodes to the skin, may improve cognition and behavior in cases of dementia. The purpose of the review^32^ (2003) was to determine the effect and safety of TENS for treating dementia, and also the variation in treatment parameters. Nine RCTs were selected, among which only three could be included in the meta-analysis. The following results were found (comparison: TENS versus placebo):

- Delayed eight-word recall, face recognition and motivation: improvement in measurements (four trials) right after treatment;
- Other neuropsychological and behavioral measures: no difference between groups immediately after treatment or after six weeks.

The limited data presented in this study did not allow any definite conclusions on the possible benefits of this intervention. For further details, refer to the original abstract, available at: http://onlinelibrary.wiley.com/doi/10.1002/14651858.CD004032/abstract.

#### 22. Validation therapy 

Validation therapy is a form of therapy using specific techniques based on acceptance of the reality and personal truth of another person’s experience. The objective of the review^33^ (2003) was to evaluate the effect of this intervention for people with cognitive impairment or dementia. Three RCTs (116 participants), showed the following results:

- Validation therapy versus usual care: validation therapy was favored in relation to behavior (one study, six weeks of treatment; MD -5.97, 95% CI -9.43 to -2.51, P = 0.0007);
- Validation therapy versus social contact: validation therapy was favored in relation to depression (one study, 12 months of intervention; MD -4.01, 95% CI -7.74 to - 0.28, P = 0.04).

There were no other statistically significant differences between validation and social contact or between validation and usual therapy. For further details, refer to the original abstract, available at: http://onlinelibrary.wiley.com/doi/10.1002/14651858.CD001394/abstract.

#### 23. Snoezelen stimulation 

Snoezelen consists of multi-sensory stimulation of the primary senses of sight, hearing, touch, taste and smell through use of lighting effects, tactile surfaces, meditative music and the odor of relaxing essential oils. The review^34^ (2002) aimed to examine the effect of Snoezelen on dementia patients and their caregivers. Two trials (246 subjects) were included, but they could not compound a meta-analysis. The results are shown below (comparison: Snoezelen versus control):

- Behavior, mood and communication/interaction: no effects from session-based Snoezelen program or 24-hour integrated Snoezelen care over the short or long term;
- Cognition: no effects from session-based Snoezelen program over the short or long term.

There was no evidence to show that Snoezelen was efficacious for treating dementia. For further details, refer to the original abstract, available at: http://onlinelibrary.wiley.com/doi/10.1002/14651858.CD003152/abstract.

#### 24. Subjective barriers for preventing wandering 

Wandering is a frequent behavioral trait among people with dementia, and it may put them at risk. The review^35^ (2000) aimed to assess the effect of subjective exit modifications (visual and other selective barriers, such as mirrors, camouflage and grids/strips of tape) on the wandering behavior of cognitively impaired people. No RCTs or controlled trials were found and, in addition, other studies were considered unsatisfactory. Therefore, there was no evidence that subjective barriers prevented wandering among cognitively impaired people. For further details, refer to the original abstract, available at: http://onlinelibrary.wiley.com/doi/10.1002/14651858.CD001932/abstract.

## DISCUSSION

This review of systematic reviews compiled through the Cochrane library focused on non-pharmacological interventions for treating cognitive impairment or dementia, regardless of etiology. It was not our primary objective to assess specific interventions for a given type of dementia but, rather, to present the evidence from up-to-date Cochrane reviews on dementia in general. Despite the major importance of this condition, there was almost no high-quality evidence for any of the outcomes proposed by the systematic reviews included. The primary studies presented limited methodological quality and other limitations, such as small sample sizes, lack of reporting of adverse effects and short-term measurement of outcomes. Consequently, the authors of these systematic reviews were unable to put forward any strong recommendations for clinical practice.

Three other previous reviews of systematic reviews^37^^,^^38^^,^^39^ aimed to evaluate interventions for treating dementia. The most recent of these^37^ compiled any systematic reviews that evaluated the effectiveness of non-pharmacological interventions on behavioral disturbances in cases of dementia. The authors’ conclusions were similar to ours. The other review^38^ focused on any kind of intervention to delay functional decline in cases of dementia. Regarding non-pharmacological interventions, the authors found only low-quality evidence relating to physical exercise and dyadic interventions. The earliest review^39^ found evidence suggesting that hand massage/gentle touch, music or music therapy and physical exercise were effective.

Differently from the others, our review only included Cochrane systematic reviews: these are developed based on rigorous explicit methods. Another important point is that we included all the outcomes proposed in the systematic reviews that were included, which was not the case for other recent reviews^37^^,^^38^ that addressed specific outcomes (behavioral disturbances and functional decline).

The limitations of the present study relate to the poor quality of the primary studies included in the systematic reviews, which lowered the strength of evidence.

Given the low quality of the primary studies, no solid recommendations for practice could be made. Some interventions seem to bring potential benefits in relation to limited outcomes, but controlled studies with high methodological quality and adequate sample sizes are needed in order to generate sound practical conclusions. The need for well-designed studies focusing on non-pharmacological interventions is particularly important, considering the personal, familial and societal burden of dementia and considering that many pharmacological interventions might not be safe in this particular population.

## CONCLUSION

A wide range of non-pharmacological interventions has been studied in the context of cognitive impairment and dementia, and some have shown potential benefits. However, the strength of evidence derived from these studies was considered low overall, because of the methodological limitations of the primary studies. 

The 24 Cochrane systematic reviews included in this study showed that carbohydrates (glucose drink) and validation therapy may be beneficial for treating cognitive impairment. For dementia, there are potential benefits from physical exercise programs, cognitive training (alone or in association with other neuropsychological rehabilitation methods), psychological treatments, aromatherapy, light therapy, cognitive rehabilitation, cognitive stimulation, hyperbaric oxygen therapy associated with donepezil, functional analysis, reminiscence therapy, transcutaneous electrical nerve stimulation, structured aid for decision-making regarding feeding options, case management approaches, interventions from non-specialist healthcare workers and use of special care units. No benefits were found from enteral tube feeding, adjunctive therapies, acupuncture, Snoezelen, respite care, palliative care team, non-pharmacological interventions for preventing wandering or subjective barriers for preventing wandering.
